# Resource Factors Allowing People with Alcohol-addicted Parents to Overcome Their Negative Emotions: A Latent Variable Model and Content Analysis

**DOI:** 10.11621/pir2021.0203

**Published:** 2021-06-30

**Authors:** Alla S. Spivakovskaya, Anna M. Lutsenko

**Affiliations:** Faculty of Psychology, Lomonosov Moscow State University, Moscow, Russia

**Keywords:** 12-step recovery program, alcohol addiction, content analysis, guilt, latent variable modeling, resource factors, shame

## Abstract

**Background:**

People with alcohol-addicted parents are at risk of psychoactive addictions, co-dependency, and suicidal behavior. Most studies of these people are aimed at confirming the inevitability of the impact of negative childhood experiences on their lives, and thus do not seek to identify resource factors which would allow them to overcome the negative emotions they experienced.

**Objective:**

The purpose of this study was to create a model of resource factors which would allow people with alcohol-addicted parents to overcome the negative emotions they experienced.

**Design:**

The participants were 58 healthy individuals (17 men and 41 women; M=25.2; SD=4.4) whose parents were alcohol addicts (they were participants in the 12-step recovery program “Adult Children of Alcoholics”), and 50 healthy individuals (15 men and 35 women, M=24.2; SD=3.7) whose parents were not alcohol addicts. The participants completed the questionnaires “Interpersonal Guilt,” “Family Emotional Communication,” and “Coping Strategies,” and were interviewed on the resource factors which allowed them to overcome negative emotions. We used the content analysis of the interviews and latent variable modeling to analyze the questionnaires.

**Results:**

The model of resource factors (CFI=0.895, RMSEA=0.064) showed that the rules set by the parental dysfunctional family (the taboo on the expressing emotions, and external well-being) were associated with being unable to recognize current negative emotions and with avoiding problems. The ability to recognize negative emotions was connected with the participant’s willingness to accept responsibility for his/ her life. The resource factors which allowed these subjects to overcome their negative emotions included: communication with relatives and friends; keeping a diary of emotions; and participating in recovery programs.

**Conclusion:**

Our model of resource factors explains the mechanism connecting dysfunctional family rules with the resource factors and negative emotions experienced by people with alcohol-addicted parents.

## Introduction

“Alcohol use disorder is a progressive disease characterized by a pathological attraction to alcohol, the development of withdrawal symptoms when alcohol is stopped, and in advanced cases, persistent somato-neurological disorders and mental degeneration” (Ivanets & Neumann, 1988). The topic of family alcohol use disorder has been actively studied during the last 10 years in Europe and Russia, largely due to the widespread prevalence of alcohol addiction (Baikova & Merinov, 2018; Brown-Rice et al., 2018; Gridneva & Tashcheva, 2018; Litvinova, 2017; Lutsenko, 2019; Lyvers & Hayatbakhsh, 2019; McCoy & Dunlop, 2017; Park & Schepp, 2015). According to Russian and American family system psychologists, psychoactive addictions, including alcohol addiction, are a family disease (Moskalenko, 2009; Potter-Efron, 2014; Woititz, 1983). The likelihood of alcohol addiction is five times higher for a person whose parents were alcohol addicts than for a person from a healthy family, and it is often impossible to live with a patient with alcohol addiction without emotional involvement in this problem (Haverfield & Theiss, 2016; Lukashyk & Filippova, 2015).

A dysfunctional family rule is often imposed in such families: “Do not talk, do not trust, do not feel.” (Tuchina et al., 2019; Woititz, 1983). This rule is meant to prevent a family member from talking about his own feelings and discussing family problems with a psychologist. The taboo on expressing negative emotions in dysfunctional families can lead to the onset of alexithymia, suppression, and experiential avoidance in adulthood (Moskalenko, 2009).

J. Woititz has argued that people with alcohol-addicted parents have a desire for external well-being and a tendency to make their expression of negative emotions taboo. (Woititz, 1983). External well-being means distrust and hostility towards people and, at the same time, the desire to make a good impression in communication. Woititz ascribed the following personal characteristics of people whose parents suffered from alcohol addiction: low self-esteem; a tendency to procrastination; a tendency to experience guilt and shame; and difficulties in establishing and maintaining friendships and family relationships. People with alcohol-addicted parents are at risk of psychoactive addictions, co-dependency, and suicidal behavior (Merinov & Shustrov, 2011; Tuchina et al., 2019). However, most of the participants in these studies were psychoactive addicts and lived with their alcohol-addicted parents. Thus these studies could not allow the identification of the personal characteristics of healthy people with alcohol-addicted parents, and the resource factors which would allow these people to overcome negative emotions (Hall & Webster, 2007; Merinov & Shustrov, 2011; Tuchina et al., 2019; Woititz, 1983).

The focus of our research was to study the emotional characteristics of mentally healthy individuals whose parents were alcohol addicts, and to identify the resource factors which would allow them to overcome their negative emotions. These people were able to overcome their negative emotions and, in some cases, help their parents overcome alcohol addiction, so studying their experience in dealing with family problems can help to understand the behavioral mechanisms that contribute to overcoming addiction.

J. Woititz, A.V. Merinov, and other researchers have emphasized the tendency to experience feelings of guilt and shame as an important factor hindering the ability of persons with alcohol-addicted parents to adapt (Baikova & Merinov, 2018). Their research found that the feeling of shame was considered “a negative emotion emerging as a result of the awareness of one’s own defect.” The feeling of guilt was considered “a negative emotion emerging as a result of attributing to a patient or his relatives the causality of negative events and real or imaginary misconduct.” J.P. Tangney and his students believed that the feeling of guilt is more productive than the feeling of shame, because the feeling of guilt contributes to a change in a person’s behavior and does not affect his positive perception of himself as a person (Tangney, 2004).

U. Orth studied families with depressed patients (Orth, Berking, & Burkhardt, 2006). He relied on Tangney’s concepts and wrote that the feeling of guilt can be associated with a patient’s acceptance of responsibility for his/her life, and with searching for social support, as opposed to the feeling of shame, which can be associated with the avoidance of the problems and the intensification of the taboo on expressing emotions. However, Orth’s investigation of the association between dysfunctional family rules, negative emotions (the feelings of guilt and shame), and the strategy of avoidance of problems was based only on data from patient interviews. We used quantitative data to create the latent variable model of resource factors which allow negative emotions to be overcome.

R.T. Potter-Efron studied the resource factors that allow people with alcohol-addicted parents to overcome their negative emotions and described a cycle of recurring emotions and behavior in these families (Potter-Efron & Potter-Efron, 1989). He wrote that since the awareness of one’s own defects leads to feelings of guilt and shame, the alcohol addict wants to avoid these emotions; therefore, he or she begins to drink, and then feels even more defective. In this cycle, family members play an important role because they can either support or impede the alcohol behavior. For example, the wife and children of an alcohol addict may not allow the patient to fulfill family roles and feel necessary to the family; such an approach will help confirm the patient’s conviction of his defectiveness. The Potter-Efron’s cycle does not imply access to resource factors, since a person who has fallen into the cycle will plunge more and more deeply into it. This approach does not include possible ways out of alcohol addiction based on the family’s resource factors.

M. Jarvinen, however, did explore possible ways out of alcohol addiction based on the family’s resource factors. She identified three resource factors in families with alcohol-addicted members: 1) communication with healthy relatives and friends; 2) hobbies; and 3) the ability to make positive plans for the future (Jarvinen, 2015). However, her study lacked a control group, and thus did not allow the identification of specific resource factors for people with alcohol-addicted parents.

J. Woititz wrote that there are differences between the behavioral interventions that can help adults whose parents were alcohol addicts to become healthy, versus behavioral interventions that can help people from healthy families. For example, communication with relatives may be the effective resource factor for healthy families, but lead to suicidal behavior in adults whose parents were alcohol addicts (Woititz, 1983). The participants in Woititz’s study were psychoactive addicts and could use resource factors specific to alcohol addicts. We decided to include the content analysis of interviews with our subjects in our research, in order to check whether the factors we identified in the quantitative research were specific to people with alcohol-addicted parents. The control group was used only for the qualitative part of the study because we wanted to compare the resource factors of the experimental and control groups.

The objective of our research was to investigate the behavioral resource factors of people with alcohol-addicted parents and create a model of resource factors which would allow them to overcome negative emotions. We hypothesized that: 1) the taboo on the expression of emotions experienced by people with alcohol-addicted parents is associated with the tendency to external well-being and the impossibility of recognizing shame and guilt; 2) shame is associated with the avoidance of the problems and the intensification of the taboo on expressing emotions; 3) guilt is connected with the participant’s acceptance of responsibility for his/her life; and 4) there are differences between the behavioral interventions that can help adults whose parents were alcohol addicts to be healthy, versus the behavioral interventions that can help people from healthy families.

## Methods

### Participants

The research was conducted by the authors of this article in Moscow in the period from January to May 2019. We are clinical psychologists who specialize in family system psychotherapy.

The study participants were 58 healthy individuals (17 men and 41 women; M=25.2; SD=4.4) whose parents were alcohol addicts (they were participants in the 12-step recovery program “Adult Children of Alcoholics”), and 50 healthy individuals (15 men and 35 women; M=24.2; SD=3.7) whose parents were not alcohol addicts. All participants were between 18 and 35 years old. Seventy-nine people (39 people from experimental and 40 people from control group) had higher education; 15 people (9 people from experimental and 6 people from the control group) had secondary specialized the education; and 14 were students (10 people from the experimental and 4 from the control group).

The inclusion criteria for the experimental group were: 1) one of the participant’s parents was an alcohol addict and was treated for this disorder throughout his/her life; 2) this parent lived with our participant and took part in his/her upbringing; and 3) the symptoms of parental alcohol addiction occurred when the participants in this research were between 6 months and 5 years old. The exclusion criteria for participation were: children from 0 to 18 years old; patients with mental disorders; patients with psychoactive addictions; and patients who were not able to sign informed consent. All participants did not have mental disorders and did not suffer from alcohol or other psychoactive addictions.

We used the Mini-Mental State Examination (MMSE) and classical pathopsychological techniques (“Pictogram,” 10 words, filling in words missed in the text, “Classification of objects,” and “Interpretation of proverbs”) to diagnose and exclude mental disorders. The participants’ self-reports and the Structured Clinical Interviews were used to diagnose and exclude alcohol or other psychoactive addictions. The Mini-Mental State Examination (MMSE) is a 30-point questionnaire that is used to examine cognitive functioning, including orientation, attention, calculation, ability to follow simple commands, ability to understand language, and recall. Six participants refused to participate in the survey, and four participants in the experimental group and two participants in the control group were excluded because they were found to have impaired cognitive functioning in the Mini-Mental State Examination (MMSE).

### Procedure

People whose parents were alcohol addicts were recruited from the recovery program “Adult Children of Alcoholics” and through the social network group “Adult Children of Alcoholics.” “Adult Children of Alcoholics” is the 12-step recovery program that supports people whose parents were alcohol addicts. Th is program has been open in Moscow since 1994. The program includes attending open and closed meetings, independent step-by-step work (following the prescribed 12 steps under the guidance of a mentor), and lectures. The main goal of “Adult Children of Alcoholics” is to create a safe space where adults whose parents were alcohol addicts can freely and constructively share their stories.

The controls in our study (people whose parents were not alcohol addicts) were recruited through social networks. Psychologists talked with the participants about their health and families and diagnosed their preservation of cognitive functions before including them in this research (Lebow, 2017).

The people whose parents were alcohol addicts completed the questionnaires and were interviewed on the topic of the resource factors which allow them to overcome their negative emotions. The controls were interviewed on the topic of resource factors which allow them to overcome their negative emotions. In the interviews, the participants spoke about themselves, their families, and their own emotional experiences. The control group was used only for the qualitative part of the study. We used face-to-face contact between the respondent and the researcher for the interviews; each interview was conducted for 50 minutes to 2 hours.

### Measures

We used the “Family Emotional Communication Questionnaire,” “The Interpersonal Guilt Questionnaire,” and the “Coping Strategies” questionnaires. All were in the Russian language.

The “Family Emotional Communication Questionnaire” consists of 30 statements (Kholmogorova et al., 2016). The Cronbach’s alpha coefficients for the individual scales of the questionnaire varied from a minimum value of 0.607 for the family perfectionism scale, up to the maximum values of 0.787 for the scales of external well-being, and 0.712 for the taboo on expressing emotions. This result indicates good internal consistency. All 30 points of the questionnaire showed even better consistency: Cronbach’s alpha was 0.807 (Kholmogorova et al., 2016). This questionnaire is aimed at diagnosing the dysfunctional family beliefs of the adult patient’s parents. Dysfunctional cognition was measured with three subscales: 1) a taboo on expressing emotions (six statements); 2) external well-being, or the desire of the family to hide, not see, and not reveal their problems (three statements); and 3) family perfectionism, which indicates very high standards for family members (three statements). Answers were measured on a 4-point scale (0 = not at all; 1 = no; 2 = maybe; 3 = yes).

“The Interpersonal Guilt Questionnaire” consists of 45 statements (Connor & Berry, 1997). Vasileva and Korotkova translated and established the procedure for standardizing the scales (Vasileva & Korotkova, 2004). They reported an internal consistency of 0.89 (Cronbach’s alpha) for the guilt of responsibility; 0.83 for the guilt of separation; 0.81 for survivor guilt; and 0.74 for the feeling of shame (Connor & Berry, 1997). This questionnaire is aimed at diagnosing actual guilt and shame. The feeling of guilt was measured with three subscales: 1) the guilt of responsibility; 2) the guilt of separation; and 3) survivor guilt. The feeling of shame was measured with one subscale. Answers were measured on a 4-point scale (0 = not at all; 1 = no; 2 = maybe; 3 = yes).

“The Coping Strategies” questionnaire (Lazarus & Folkman, 1988) consists of 50 statements. Cronbach’s alpha was analyzed for all factors proposed, resulting in the following coefficients: 1) acceptance of responsibility (α = 0.77); 2) avoidance (α = 0.66); and 3) search for social support (α = 0.86); these results indicate a good internal consistency (Krukova, 2001). Krukova translated and established the procedure for standardizing the scales (Krukova, 2001). We used this questionnaire to measure family resource factors in individuals whose parents were alcohol addicts. The results pertained to eight subscales. Three subscales were used for the cognitive-behavioral model of resource factors because these subscales correlated with guilt and shame: 1) search for social support (six statements); 2) acceptance of responsibility (four statements); and 3) escape-avoidance (eight statements). The answers were measured on a 4-point scale (0 = not at all; 1 = no; 2 = maybe; 3 = yes).

### Data Analysis

We applied content analysis of the transcripts of the interviews with the participants of the rehabilitation program “Adult Children of Alcoholics” and with the controls. Our data collection came from each participant being interviewed and answering the question: “What helped you overcome negative emotions associated with family dysfunction?” We identified and highlighted semantic units of analysis, grouped units by topics (eight categories related to the family resource factors were described and interpreted), calculated the percentage of responses, and interpreted the data obtained (Riffe, 2019; Ylanovski, 2007).

### Statistical Analysis

The EQS 6.2 Structural Equations Program Manual statistics program was used for quantitative analyses of the structural model of family resource factors (Bentler, 2006). We used latent variable modeling for data analysis. Estimations were based on the covariance matrix and the maximum likelihood method. Fixation of factor loadings was used as the scaling method. Five fit indices assessed model fit: IFI= Incremental Fit Index; CFI= Comparative Fit Index; SRMR = standardized root mean square residual; RMSEA = root mean square error of approximation; and the Confidence Interval of RMSEA. Values less than or equal to 0.05 for RMSEA, values less than or equal to 0.08 for SRMR, and values greater than or equal to 0.80 for IFIand CFIindicated good fit.

Degree of freedom and χ2 statistics were also used to judge the fit of the model. The χ2-distribution with n degrees of freedom is the distribution of a sum of the squares of independent standard normal random variables. The ratio of χ2 statistics to the number of degrees of freedom df should not be greater than 2. We used the k-factor-corrected Satorra-Bentler scaled (not adjusted) test statistic to evaluate model fit in small samples. We used the Bonferroni correction to control for multiple tests.

Correlation statistical analysis was conducted using SPSS statistics (Version 22.0). We used the Pearson correlation coefficient to measure the linear correlation between negative emotions experienced by people with alcohol-addicted parents and their resource factors, between the rules set by the parental dysfunctional family and the resource factors, and between the rules set by the parental dysfunctional family and negative emotions. The Pearson correlation coefficients ranged from −1 to 1. Values of less than 0.3 indicated a low correlation; values greater than or equal to 0.3 indicated significant correlations.

## Results

### The Pearson Correlation Coefficient

We used the Pearson correlation coefficient to measure the linear correlation between negative emotions experienced by people with alcohol-addicted parents (the feelings of guilt and shame, which was measured using the “The Interpersonal Guilt Questionnaire”), the rules set by the parental dysfunctional family (taboo on expressing emotions, external well-being, and family perfectionism, which were measured using the “Family Emotional Communication Questionnaire”), and the family resource factors (search for social support, acceptance of responsibility, and escape-avoidance, which were measured using “The Coping Strategies”). In the preliminary analyses, the means, standard deviations, and the Pearson correlation coefficients of indicators were estimated, as seen in *Table 1*.

**Table 1:** Means, Standard Deviations and Pearson’s r of indicators used to measure Taboo on expressing emotions, External well-being, Family perfectionism, Guilt, Shame, Search for social support, Acceptance of responsibility, and Escape-avoidance (N=58)

Scales	M	SD	1	2	3	4	5	6	7
1. Taboo on expressing emotions (6 Indicators)	1.75	0.6	–						
2. External well-being (3 Indicators)	1.91	0.83	0.22	–					
3. Family (3 Indicators) perfectionism	1.84	0.81	0.06	0.42*	–				
4. Guilt (3 Indicators)	1.90	0.45	–0.27	–0.04	0.00	–			
5. Shame (1 Indicator)	1.55	0.73	–0.08	–0.30*	–0.02	0.18	–		
6. Search (6 Indicators) for social support	2.04	0.55	0.05	–0.10	0.06	–0.27	–0.09	–	
7. Acceptance (4 Indicators) of responsibility	1.77	0.56	–0.08	–0.32*	–0.18	0.17	0.43*	0.06	–
8. Escape-(8 Indicators) avoidance	1.70	0.44	0.14	–0.19	–0.04	–0.01	0.34*	0.21	0.25

Note* p<0.05

To build the structural model of resource factors, which would allowing people with alcohol-addicted parents to overcome their negative emotions, we used only those coping strategies that correlated with the feelings of guilt or shame. Therefore, we excluded the following strategies from the model as unrelated to the emotions we were studying: 1) confrontation (the correlation between confrontation and guilt = -0.12; confrontation and shame = 0.10); 2) distance (the correlation between distance and guilt = 0.13; distance and shame = 0.12); 3) self-control (the correlation between self-control and guilt = 0.10; self-control and shame = –0.09); 4) planning a solution to a problem (the correlation between planning a solution to a problem and guilt = –0.10; planning a solution to a problem and shame = –0.02); and 5) positive revaluation (the correlation between positive revaluation and guilt = 0.02; positive revaluation and shame = –0.10).

The Pearson’s r results showed that the taboo on expressing emotions experienced by people with alcohol-addicted parents was associated with external well-being, and negatively associated with the feeling of guilt. External well-being indicates the desire of the family to hide, rather than see and reveal their problems. External well-being was positively associated with family perfectionism and a tendency to avoid responsibility, and negatively associated with the feeling of shame. The feeling of guilt was negatively associated with the search for a social support coping strategy. The feeling of shame was associated with the acceptance of responsibility for their lives and escape-avoidance. Escape-avoidance was associated with the acceptance of responsibility and with the search for social support.

### A Latent Variable Model of the Resource Factors

**Figure 1.**
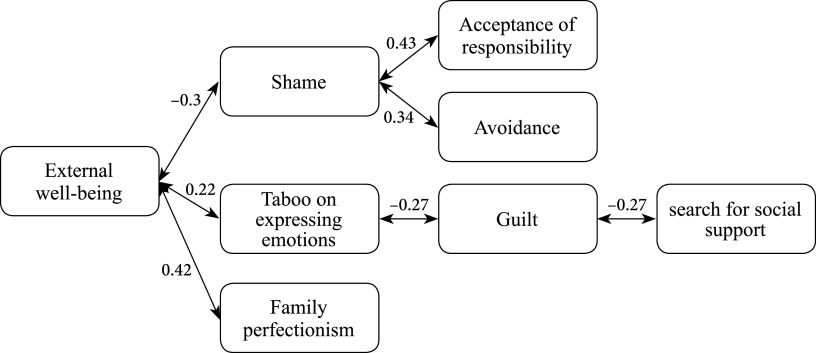
The structural model of resource factors and negative emotions experienced by people with alcohol-addicted parents

Our structural model of the resource factors which allow people with alcohol-addicted parents to overcome their negative emotions, was created based on our correlation analysis (*Figure 1*).

Our structural model of resource factors and negative emotions was tested, as seen in *Table 2*. We used latent variable modeling to test our first model. This model did not fit well with the data, so we excluded three factors (escape-avoidance, acceptance of responsibility, and search for social support) to improve it. Model 2 thus consisted of five factors: external well-being; family perfectionism; taboo on expressing emotions; and the feelings of guilt and shame. The suitability indices of the model 2 can be considered good.

**Table 2:** Indicators for Measurement Models (N=58)

Models	X^2^	df	IFI	CFI	SRMR RMSEA	90% confidence interval of RMSEA
Model emotions 1: Parental and coping influence, strategies	796.696	518	0.538	0.503	0.119 0.097	0.083 to 0.109
Model emotions 2: Parental influence and	123.619	100	0.904	0.895	0.096 0.064	0.000 to 0.098

*Note. IFI= Incremental Fit Index; CFI= Comparative Fit Index; SRMR = standardized root mean square residual; RMSEA = root mean square error of approximation. p<0.05*.

### The Content Analysis of the Interviews

The control group was used only for the qualitative part of the study so that we could compare the resource factors of the experimental and control groups. Content analysis of the interviews with both groups consisted of data collection (each participant answered the question: “What helped you overcome negative emotions associated with family dysfunction?”), highlighting semantic units of analysis, calculating the percentages of the various responses, and interpreting the data obtained. The subjects were asked to describe how eight categories related to the family resource factors helped them overcome the consequences of family dysfunction, as seen in *Table 3*.

**Table 3:** Content analysis of the family resource factors in experimental and control groups

Categories	Experimental group (N=58)		Control group (N=50)	
	Number of responses	Percentage of responses	Number of responses	Percentage of responses
keeping a diary	8	13.8	4	8
participation in a rehabilitation program	12	20.6	0	0
communication with friends	7	12.1	16	32
communication with grandparents	10	17.2	10	20
sport	3	5.2	12	24
illness	8	13.8	3	6
escape-avoidance	4	6.9	2	4
faith	6	10.3	3	6

#### Keeping a diary

Eight people of the experimental group and four people of the control group kept diaries of their thoughts and emotions in the family context. They said that keeping a diary helped them to understand their emotions and, in some cases, to overcome feelings of guilt and anger towards alcohol-addicted parents. People whose parents had alcohol addiction mentioned this type of family resource factors more often than the control group because people from the experimental group kept diaries while participating in the rehabilitation program.

Some examples: “The diary helps me to understand myself, my emotions, and my family. I usually write down what I feel in my family; it helps me to analyze the situation and to understand how I can help my mother and myself.” “When I was very angry with my alcohol-addicted mother in my childhood, I began to keep a diary. It helped me to express my feelings and to relieve my aggression.” “Keeping a diary helped me to understand that I do not want to live like my alcohol-addicted parents. I do not want to drink, to fight, to break dishes, to beg for money from relatives. I decided to completely abandon alcohol.”

#### Participation in a rehabilitation program

People from the experimental group mentioned this resource factor because they took part in the rehabilitation program “Adult Children of Alcoholics” and believed that this program could help them. People whose parents had alcohol addiction paid attention to the opportunities of the rehabilitation program: they could speak about their family problems in the program, find new friends, understand their feelings, and try to find their own resource factors. In the rehabilitation program, the participants come to understand that they are not alone and that they can overcome their negative emotions.

Some examples: “Participation in the 12-step rehabilitation program “Adult Children of Alcoholics” helps me to understand that I am not alone, my problem is not unique, and I can overcome the consequences of family problems. I usually visit open and closed meetings of the program and I feel safe there.” “I understand that in my family there is a problem. Before my participation in the 12-step rehabilitation program, I had never thought that it is not normal that my parents do not take care about me and my sister, that I cannot invite friends to visit me in my flat. I thought that it was normal, but now I can explain the problems of my family and I believe that I can overcome them.”

#### Communication with friends

This type of resource factor was mentioned by both the control and experimental groups. People whose parents had alcohol addiction said that friends helped them to understand moral norms and caretaking behavior. In some cases, friends’ parents became the ideal models of family communication. Two participants mentioned that friends helped them to find the rehabilitation center for their parents.

Some examples: “I need somebody to help me accept my feelings and discuss my family problems. My ex-boyfriend was a drug addict; I tried to save our relationship and discuss my family problems with him. Nowadays I know that alcohol and drug addictions are dangerous diseases and I try to communicate with healthy people. My friends help me to feel that I am not alone and together we can help my father to become healthy.” “My friend helped me find the rehabilitation center for my mother. My friend taught me how to cook because my mother had alcohol addiction.”

#### Communication with grandparents

Both the control and the experimental groups mentioned this type of resource factor. People whose parents had alcohol addiction usually reported that healthy grandparents were directly involved in their upbringing and took care of them. Some of the participants wanted to be like their grandparents. Grandparents from such families were strict with their grandchildren, because they did not want a recurrence of alcohol problems in the families of their grandchildren.

Some examples: “My grandmother was directly involved in my upbringing. I know that my parents are patients with alcohol addiction. However, I do not have psychoactive addictions because my strict grandmother took care of me in my childhood. I remember my grandmother and understand that I have an opportunity to be healthy and to have healthy children.” “My father was an alcohol addict. When I think about the image of a man, I think about my grandfather. He was strict, but everyone respected him, he did not avoid accepting responsibility for the whole family. I want to be like my grandfather in my own family.”

#### Sport

People whose parents had alcohol addiction noticed that team sports helped them to keep a healthy lifestyle, to communicate with friends from healthy families, and to feel safe. Some participants came to sports because they wanted to protect themselves and their younger siblings from parental aggression.

Some examples: “I enrolled in the wrestling team to be able to protect myself in the family. There I made friends from healthy families. Sport has protected me from alcohol.”

#### Illness

People with alcohol-addicted parents used this coping strategy more often than people from the control group. People whose parents had alcohol addiction wrote that their illness, in some cases, helped their parents to stop drinking and begin to try to take care of their children. Illness also helped people with alcohol-addicted parents to stay home and postpone visits to parents with whom they did not want to communicate.

Some examples: “When I have a disease, I don’t think about my parental alcohol family; I need to become healthy. Illness helps you to understand that you value life and you can forgive your parents.” “When I try to think about my childhood and parents, I get a headache. I think my headache prevents me from remembering my childhood.”

#### Escape-avoidance

Some participants in both the control and experimental groups reported that the best way to solve the problem was to ignore it. For example, some people whose parents had alcohol addiction completely cut off communication with their parents, whose behavior they did not like.

Some examples: “I don’t communicate with my parents. I don’t visit them because I don’t want to see that they drink alcohol. They are very weak; I don’t want to have such parents.”

#### Faith

Some participants thought that the best way to deal with a parental problem was to rely on God’s help and pray. Some of them wanted to give up the responsibility for their lives and the lives of their families and shift all responsibility to God.

Some examples: “I prayed that my father would stop beating my mom and drinking alcohol. Father became less aggressive after a rehabilitation course. God heard me, so He is on my side.” “When my mother came home after a noisy party with alcohol and went to bed, I could only pray that our neighbors and my friends would not know about her behavior. Only God could save me from the feeling of shame.”

## Discussion

The sample in our study was unusual, because researchers usually study psychoactive addicts with alcohol-addicted parents, but we studied healthy individuals whose parents had alcohol addiction. These people were able to overcome the consequences of family problems and, in some cases, help their parents to overcome alcohol addiction, so studying their experience can help us to understand the behavioral mechanisms that contribute to overcoming addiction. We created a model of the resource factors which would allow healthy individuals whose parents had alcohol addiction to overcome their negative emotions. This model expands the understanding of the characteristics of negative emotions and the resource factors of these people for use in clinical practice.

Our model of resource factors explains the mechanism which connects the dysfunctional family’s rules (the taboo on the expressing emotions and external wellbeing) with the resource factors and negative emotions experienced by people with alcohol-addicted parents. The results showed that the resource factors allowing them to overcome negative emotions included: communication with relatives and friends; keeping a diary of emotions; and participating in recovery programs.

The importance of this study lies in the fact that it shows the relationship between parental dysfunctional rules and the coping strategies of children in the family who became mentally healthy people. The data we obtained on the types of resource factors can be used in clinical practice to work with the negative emotions of people with alcohol-addicted parents.

Our results showed that the negative emotions experienced by people with alcohol-addicted parents (the feelings of guilt and shame) are associated with the dysfunctional family’s rules (the taboo on the expressing emotions and stress on external well-being). J. Woititz wrote that usually there is a dysfunctional family rule in families with alcohol-addicted parents: “Do not talk, do not trust, and do not feel.” (Woititz, 1983). This rule forbids members of the dysfunctional families to express emotions. However, she studied alcohol addicts whose parents were alcohol addicts, and only analyzed interviews with them. In our research, where the participants were not alcohol addicts, but the participants’ parents were, we found similar results.

The taboo on expressing emotions in families with members addicted to alcohol is associated with the tendency to external well-being in these families (r = 0.22). A.V. Merinov wrote that people whose parents were alcohol addicts have a tendency toward suicidal and co-dependent behavior, and usually use emotion-focused coping strategies (Baikova & Merinov, 2018). He explained their behavior by a tendency to external well-being and a lack of the ability to speak about their family problems.

We expected that shame would be associated with the avoidance of the problems and the intensification of taboo on expressing emotions, and guilt would be connected with the acceptance of responsibility for the respondent’s life. According to J.P. Tangney (2004) and her students, the feeling of guilt is more productive than the feeling of shame, because guilt contributes to motivating a change in a person’s behavior and does not affect his positive perception of himself as a person. However, in our study the feeling of guilt was not connected with the acceptance of responsibility for the respondent’s life. The results of the content analysis of the interviews showed that the feeling of guilt was not seen as an emotion leading to accessing the family’s resources. The participants said that the feeling of guilt prevented them from searching for social support when they needed it, and did not allow them to accept responsibility for their lives.

The results showed that the inability to recognize shame was associated with problem avoidance and the intensification of the taboo on expressing emotions. R.T. Potter-Efron described a cycle of recurring negative emotions and behavior in families with members addicted to alcohol (Potter-Efron, 2014). He wrote that the awareness of one’s own defects leads to feelings of shame; since the alcohol addict wants to avoid these emotions, he/she begins to drink and feels more defective. The Potter-Efron’s cycle does not include access to resource factors, since a person who has fallen into the cycle will plunge more and more deeply into it. Our research shows that there is an opportunity to exit the cycle through the recognition of a feeling of shame and taking responsibility for this feeling. The ability to recognize shame was connected with the participant’s acceptance of responsibility for his/her life.

There are differences between the behavioral interventions that can help adults whose parents were alcohol addicts to be healthy versus the behavioral interventions that can help people from healthy families. These data expand the list of resource factors for this category of individuals that family system psychotherapists obtained in previous studies (Drapkin et al., 2015; Kim & Ko, 2019). Adults whose parents were alcohol addicts use communication with grandparents and friends, keeping a diary of emotions, participating in recovery programs, and taking classes in sports. The participants also used three dysfunctional resource factors (illness, escape-avoidance, and faith) to temporarily cope with their emotions. The study participants called these three factors dysfunctional, because they helped to cope with the problem temporarily, but did not change the situation in the long term. People whose parents were not alcohol addicts did not mention participation in rehabilitation programs, and rarely resorted to the use of illness and avoidance of responsibility.

We expect that these results should have some benefit for creating rehabilitation programs for people with alcohol-addicted parents, because this study clearly identified the resource factors these people can access.

## Conclusion

In this study, we created a structural model of resource factors which would allow people with alcohol-addicted parents to overcome their negative emotions. This model explained the mechanism connecting the family’s dysfunctional rules with the resource factors and the current emotions experienced by people whose parents were alcohol addicts. The family rules of the parental dysfunctional family (the taboo on expressing emotions and external well-being) were associated with the difficulty of recognizing the current shame and guilt experienced by people whose parents were alcohol addicts. The ability to recognize shame was connected with the acceptance of responsibility for the participant’s life. The family resource factors of adults with alcohol-addicted parents included: communication with grandparents and friends, keeping a diary of emotions, participating in recovery programs, and taking sports classes.

It would be useful to study people whose parents were drug addicts and to compare them with people whose parents had alcohol addiction. We expect that the models of resource family factors for families with members addicted to alcohol and for families with drug-addicted members would not differ significantly. If this expectation is true, the structural model of family resource factors in patients with alcohol addiction could be useful for describing the mechanisms connecting the dysfunctional family’s rules with resource factors with their current emotions experienced by families with psychoactive addicted patients.

## Limitations

There are some limitations to this research. The sample was not large, and that may be a serious limitation, because in small samples, many structural models may be accepted (not rejected statistically) that would not hold up in larger samples. Thus, we can only characterize the correlations revealed in the study as trends. In further research, it is necessary to test our model and other variants of the models of resource factors on a large sample (n> 100). We did not test for gender differences in the study, because 95% of the “Adult Children of Alcoholics” program members are women. This can also be a serious limitation, because avoidant coping strategies often differ between men and women (Panayiotou et al., 2017).

The experimental group included only healthy individuals whose parents were alcohol addicts, and who were the participants in the 12-step recovery program “Adult Children of Alcoholics.” We did not study people who were not the participants of the 12-step rehabilitation program and who were psychoactive addicts; these groups of people do not want to use participation in recovery programs as a primary resource factor. We expect that these groups of people may use other resource factors to overcome negative emotions, such as alcohol, exercising self-control, and isolation.
